# Impact of a personalized, strike early and strong lipid-lowering approach on low-density lipoprotein-cholesterol levels and cardiovascular outcome in patients with acute myocardial infarction

**DOI:** 10.1093/ehjcvp/pvaf004

**Published:** 2025-01-24

**Authors:** Giuseppe Patti, Luca Cumitini, Manuel Bosco, Alessandra Marengo, Domenico D'Amario, Marco Mennuni, Martina Solli, Leonardo Grisafi

**Affiliations:** Department of Translational Medicine, University of Eastern Piedmont, Via Solaroli 17, 28100 Novara, Italy; Division of Cardiology, Maggiore della Carità Hospital, Corso Mazzini 18, 28100 Novara, Italy; Department of Translational Medicine, University of Eastern Piedmont, Via Solaroli 17, 28100 Novara, Italy; Department of Translational Medicine, University of Eastern Piedmont, Via Solaroli 17, 28100 Novara, Italy; Department of Translational Medicine, University of Eastern Piedmont, Via Solaroli 17, 28100 Novara, Italy; Department of Translational Medicine, University of Eastern Piedmont, Via Solaroli 17, 28100 Novara, Italy; Division of Cardiology, Maggiore della Carità Hospital, Corso Mazzini 18, 28100 Novara, Italy; Department of Translational Medicine, University of Eastern Piedmont, Via Solaroli 17, 28100 Novara, Italy; Division of Cardiology, Maggiore della Carità Hospital, Corso Mazzini 18, 28100 Novara, Italy; Division of Cardiology, Maggiore della Carità Hospital, Corso Mazzini 18, 28100 Novara, Italy; Division of Cardiology, Maggiore della Carità Hospital, Corso Mazzini 18, 28100 Novara, Italy

**Keywords:** Dyslipidemia, Myocardial infarction, Lipid-lowering therapies, LDL-C, Strike early and strike strong, Major adverse cardiovascular events

## Abstract

**Aims:**

Considering the lack of evidence, we evaluated the impact on cardiovascular outcome of the systematic introduction in our institution of a personalized strike early and strong (SES) approach for lipid-lowering therapy (LLT) in patients admitted for acute myocardial infarction (MI).

**Methods and results:**

We retrospectively analysed data from 500 consecutive patients hospitalized across three periods: Period A (*N* = 198, January–June 2019), when the low-density lipoprotein cholesterol (LDL-C) goal was <70 mg/dL and a stepwise LLT approach was recommended; Period B (*N* = 180, January–June 2021), when the LDL-C goal was <55 mg/dL and a stepwise approach was recommended; Period C (*N* = 122, January–June 2023), when the LDL-C goal was <55 mg/dL and our SES protocol was implemented. Primary endpoints were achievement of the LDL-C goal during follow-up and 1-year incidence of major adverse cardiovascular events (MACE). Compared to the other periods, in Period C, there was a higher use of potent statins, alone or in combination with ezetimibe, and of proprotein convertase subtilisin/kexin type 9 inhibitor inhibitors at discharge. This translated into higher achievement of the LDL-C goal (83% vs. 55% in Period A and 43% in Period B; *P* < 0.001) and reduced incidence of MACE (3% vs. 12% and 11%; *P* = 0.026). MACE rates were lowest in patients with early and sustained LDL-C <55 mg/dL and in those achieving both LDL-C <55 mg/dL and ≥50% LDL-C reduction.

**Conclusion:**

The systematic introduction of a personalized, SES strategy for LLT in patients with acute MI led to greater achievement of LDL-C goal and lower risk of MACE at 1 year vs. the stepwise approach.

## Graphical Abstract

**Graphical Abstract:**
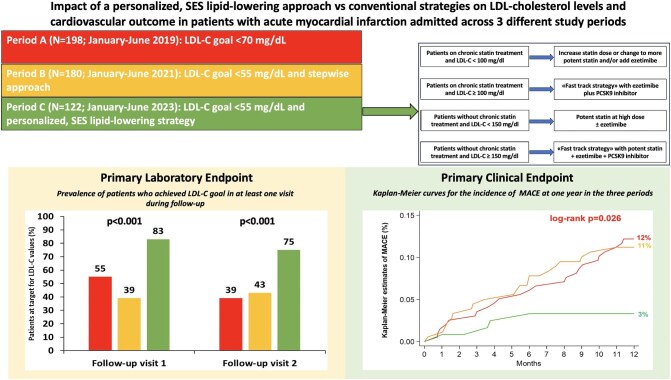
The systematic introduction in patients with acute myocardial infarction of a personalized, SES lipid-lowering approach led to greater achievement of the LDL-C goal during follow-up and to lower risk of MACE at 1 year vs. the stepwise approach. LDL-C, low-density lipoprotein cholesterol; MACE, major adverse cardiovascular events; PCSK9i, proprotein convertase subtilisin/kexin type 9 inhibitor; SES, strike early and strong.

## Introduction

The clinical management of dyslipidemias in patients experiencing an acute myocardial infarction (MI) has evolved significantly over the past decade.^1^ The former 2016 European Society of Cardiology (ESC)/European Atherosclerosis Society (EAS) guidelines for the management of dyslipidemias recommended reaching a low-density lipoprotein cholesterol (LDL-C) goal <70 mg/dL in patients at very high cardiovascular risk.^2^ Such recommendation was based on the evidence that this threshold could provide the best benefit in terms of balance between efficacy and safety of lipid-lowering therapies (LLTs). It was also suggested that statin monotherapy would have been effective in achieving this goal in the majority of these patients.^2^ The introduction of ezetimibe and, more recently, of proprotein convertase subtilisin/kexin type 9 (PCSK9) inhibitors alongside statins, has allowed to achieve in the individual patient very low LDL-C levels, especially when LLTs are given in combination. Of note, various studies have clearly demonstrated a linear correlation between on-treatment LDL-C values and reduction of major adverse cardiovascular events (MACE), with maintained clinical efficacy and no safety concerns when very low LDL-C concentrations are reached.^3–7^ Accordingly, in 2019, the ESC/EAS updated the recommendations indicating an even more stringent LDL-C goal (<55 mg/dL) in patients at very high cardiovascular risk, including those with acute MI.^8^ To achieve this lower goal, the 2019 ESC/EAS guidelines continued to support a stepwise approach for LLT, already recommended in the 2016 guidelines. This strategy includes an early initiation of a potent statin and subsequent association with ezetimibe first, and then with a PCSK9 inhibitor, if the LDL-C goal was not achieved.^2,8^ However, several real-world registries showed that the stepwise approach is associated with low percentages of patients (generally < 40%) achieving the goal of LDL-C <55 mg/dL post-acute MI.^9–17^

More recently, in European consensus documents, it has been hypothesized that a ‘strike early and strong’ (SES) approach for LLT in patients with acute MI can lead to a faster and greater LDL-C goal achievement.^18,19^ Such strategy includes the initiation, already during hospitalization, of LLTs in combination. To date, no data on clinical outcome with the use of this comprehensive and ‘more aggressive’ approach are available. The aim of the study was to evaluate the effects of a systematic application in patients with MI of a pre-determined and personalized SES strategy for LLT, aimed at optimizing the treatment during in-hospital stay or at discharge. We have specifically investigated whether the implementation of such strategy translates into a greater and faster achievement of the LDL-C goal and improves MACE-free survival at 1 year.

## Methods

### Study design

**Figure 1:**
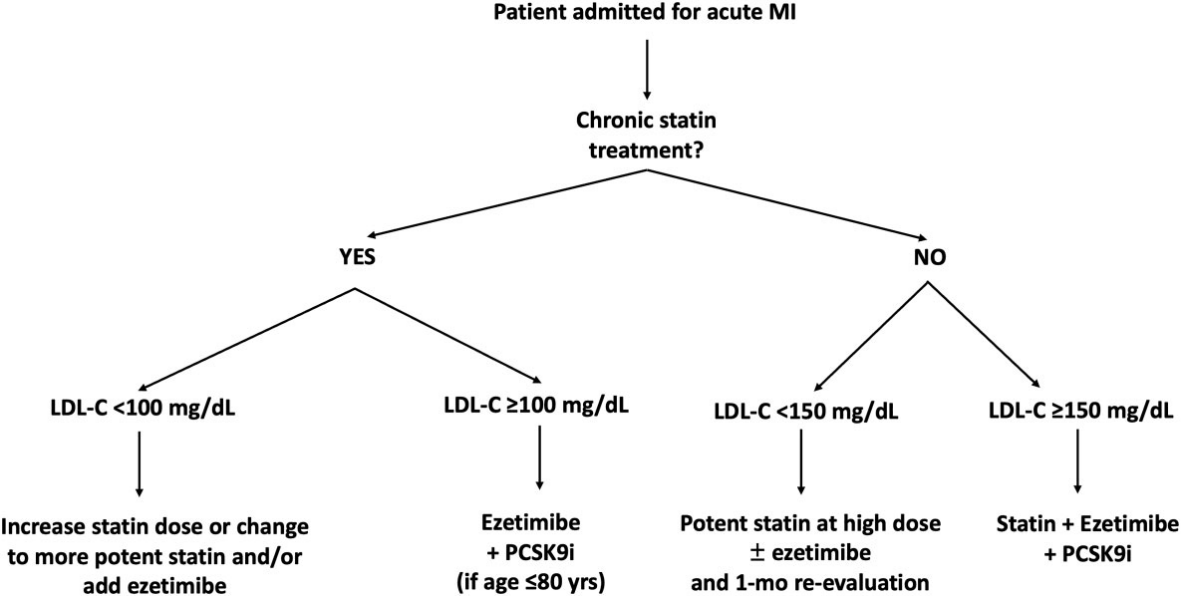
Personalized flowchart of SES for LLT given during hospitalization/at discharge in patients admitted for acute MI in Period C. Patients on chronic statin treatment and LDL-C <100 mg/dL: The required relative LDL-C reduction to achieve the goal is here < 45%; considering that each statin doubling dose gets approximately 7% of LDL-C decrease and ezetimibe is associated with 20% LDL-C reduction, these patients are treated by increasing statin dose or changing to more potent statin and/or adding ezetimibe. Patients on chronic statin treatment and LDL-C ≥100 mg/dL: In this case, the distance to the goal is ≥ 45% LDL-C decrease, and no modification of oral LLT (increase of statin dose, change of statin type, addition of ezetimibe alone) would be able to get the goal; thus, a fast-track with addition of ezetimibe + PCSK9 inhibitor is performed. Notably, the use of PCSK9 inhibitors in Italy is reimbursed by the National Health System if LDL-C is >70 mg/dL and age is ≤ 80 years. Patients without chronic statin treatment and LDL-C <150 mg/dL: The required relative LDL-C reduction is here < 65%; therefore, these patients are given oral LLT with potent statin at high dose plus ezetimibe if the distance to the goal is > 50% or with potent statin at high dose alone, if the distance to the goal is ≤ 50%. Patients without chronic statin treatment and LDL-C ≥150 mg/dL: In this case, the expected 65% LDL-C reduction achievable with high-intensity statin plus ezetimibe is inadequate to reach the recommended <55 mg/dL LDL-C goal; thus, a fast-track with potent statin plus ezetimibe and PCSK9 inhibitor (triple LLT), resulting in up to 85% LDL-C reduction, is performed. This treatment algorithm applies to all patients with very high cardiovascular risk, but for the purpose of our study, it has been considered in the setting of patients with acute MI. LDL-C, low-density lipoprotein cholesterol; LLT, lipid-lowering therapy; MI, myocardial infarction; PCSK9i, proprotein convertase subtilisin/kexin type 9 inhibitor; SES, strike early and strong.

FAST track—NOvara Therapeutic carE pathway (FAST-NOTE) is an observational, monocentric, retrospective study on consecutive patients admitted for acute MI at Maggiore della Carità Hospital in Novara. All patients had LDL-C values not at target at the time of the index hospitalization and underwent percutaneous coronary intervention during three specific periods:

- Period A: From 1 January 2019 to 30 June 2019, when patients were managed according to the 2016 ESC/EAS guidelines for dyslipidemias, indicating a goal of LDL-C < 70 mg/dL and a stepwise approach.^2^
- Period B: From 1 January 2021 to 30 June 2021, when patients were managed following the 2019 ESC/EAS guidelines, which recommended a goal of LDL-C < 55 mg/dL and a stepwise approach.^8^
- Period C: From 1 January 2023 to 30 June 2023, when in our Institution had been implemented a specific protocol of personalized and intensive LLT, prescribed during in-hospital stay or at discharge, and designed to reach as early as possible the goal of LDL-C < 55 mg/dL (SES strategy). In detail, patients are stratified and treated according to baseline risk, background chronic LLT, baseline LDL-C, % LDL-C reduction needed to achieve the recommended goal, and predictable LDL-C% reduction with available LLTs. This therapeutic protocol is described in *Figure 1*.

### Data collection

Data were obtained from medical records during the index hospitalization and at follow-up visits. Follow-up visits were performed at 1 month (visit 1), at 3–4 months (visit 2), and 1 year. Information on patient's characteristics, cardiovascular risk factors, comorbidities, previous cardiac events and interventions, echocardiographic measures, procedural features, prior LLTs, and concomitant medications at the time of the index event was collected. Data on outcome and on drug therapies were also recorded at each follow-up visit. Laboratory findings, also including the lipid profile [total cholesterol, high-density lipoprotein cholesterol (HDL-C), measured LDL-C, and triglycerides], were available at baseline (during the index hospitalization) and at visits 1 and 2. Patients who died before discharge and after discharge, but before visit 1, were excluded. The study adhered to the principles of the Declaration of Helsinki and Good Clinical Practice guidelines. The study protocol was approved by the Local Ethics Committee (approval number: CE 018/2024).

### Study endpoints

Data from patients enrolled in Period C (<55 mg/dL LDL-C goal and personalized, SES approach for LLT) were compared with those from patients of Period A (<70 mg/dL LDL-C goal and stepwise approach) and Period B (<55 mg/dL LDL-C goal and stepwise approach). Laboratory primary endpoint was the prevalence of patients reaching the LDL-C target at follow-up visits in Period C compared to Periods A and B. Clinical primary endpoint was to evaluate if the SES approach in Period C was associated with lower incidence of MACE (composite of cardiovascular death, acute MI, stroke, or unplanned coronary revascularization) at 1 year vs. Periods A and B.

The following other outcome measures were analyzed:

- Changes of LDL-C values at follow-up visits 1 and 2 vs*.* baseline in Period C compared to Periods A and B.
- Levels of LDL-C at follow-up visits 1 and 2 in Period C compared to Periods A and B.
- Independent predictors of reaching the goal of LDL-C 55 mg/dL during follow-up and of MACE at 1 year.
- MACE incidence according to achievement or not of the 55 mg/dL LDL-C goal, in combination or not with ≥ 50% LDL-C reduction at follow-up evaluations vs. baseline. Baseline LDL-C was considered the LDL-C value during the index hospitalization, regardless of whether patients were taking LLT prior to the admission.
- Incidence of MACE at 1 year according to maintenance of the LDL-C goal.
- Quantitative and qualitative changes in LLT regimens from discharge to visit 1 and from visit 1 to visit 2.

### Statistical analysis

As continuous variables were not normally distributed by the Kolmogorov—Smirnov test, they are presented as median [interquartile range]. Categorical variables are indicated as absolute numbers and percentages. Differences in continuous variables between different periods were analysed using the Kruskal–Wallis and Wilcoxon rank-sum tests. Differences in continuous variables between measurements within the same period were calculated by the Wilcoxon matched-pairs signed-ranks test. The *χ*^2^ test was utilized to compare categorical variables between different periods, and the McNemar test to compare categorical variables within the same period. Kaplan–Meier curves and log-rank test were utilized to compare the incidence of MACE at 1 year between different LLT approaches and LDL-C levels. Logistic regression analysis [expressed as odds ratios (ORs) and 95% confidence intervals (CIs)] was performed by a stepwise method to identify independent predictors of achieving LDL-C goal during follow-up. A Cox proportional hazards model [expressed as hazard ratios (HRs) and 95% CI] was applied, using a stepwise method, to identify independent predictors of MACE at 1 year. The variable ‘SES approach’ was forced into the models, and only covariates with a *P*-value < 0.05 at univariate analysis were included in the multivariate models. After testing collinearity between variables by linear regression analysis, only those variables with a variance inflation factor between 1 and 3 were considered. Statistical analyses were performed using STATA 18.0 software (StataCorp, LP, College Station, Texas) and SPSS Statistics for Windows software (IBM Corp; Version 29.0, Armonk, NY), with a *P* value < 0.05 considered significant.

## Results

### Baseline characteristics

A total of 500 patients admitted for acute MI were overall included: 198 in Period A, 180 in Period B, and 122 in Period C. Baseline characteristics are presented in *Table 1*. In the overall population, median age and prevalence of female gender were 66 years [59–76] and 27.6%, respectively, without differences between the three periods. ST-segment elevation MI was the most frequent clinical presentation, with a higher prevalence in Period C. Left ventricular ejection fraction was more elevated in Period A (53% vs. 50% in the other periods). The prevalence of diabetes was lower in Period C. The overall use of statin and ezetimibe before the admission was 20% and 1.6%, respectively, without differences between the three periods. Total cholesterol and LDL-C at baseline were comparable in the three periods.

**Table 1:** Baseline characteristics

			Period		
	Overall	A	B	C	
Variables	*N* = 500	*N* = 198	*N* = 180	*N* = 122	*P* value
Age (years)	66 [59–76]	66 [57–76]	67 [59–78]	65 [58–73]	0.20
Female gender	138 (27.6%)	45 (22.7%)	53 (29.4%)	40 (32.8%)	0.12
BMI (Kg/m^2^)	26 [24–29]	26 [24–29]	26 [23–29]	26 [24–28]	0.78
Current smokers	185 (37.0%)	58 (29.3%)	63 (35.0%)	64 (52.5%)	**<0.001**
Systemic hypertension	320 (64.1%)	128 (64.6%)	121 (67.6%)	71 (58.2%)	0.24
Diabetes mellitus	105 (21.0%)	49 (24.7%)	41 (22.8%)	15 (12.3%)	**0.022**
Chronic kidney disease	96 (19.2%)	33 (16.7%)	42 (23.3%)	21 (17.2%)	0.21
Peripheral arterial disease	51 (10.2%)	22 (11.1%)	23 (12.8%)	6 (4.9%)	0.07
Prior MI	84 (16.8%)	37 (18.7%)	31 (17.2%)	16 (13.1%)	0.43
Multivessel disease	262 (52.4%)	103 (52.0%)	97 (53.9%)	62 (50.8%)	0.86
LVEF (%)	51 [44–56]	53 [45–57]	50 [44–55]	50 [43–55]	**0.006**
Clinical presentation					
STEMI	307 (61.4%)	110 (55.6%)	107 (59.4%)	90 (73.8%)	0.004
NSTEMI	193 (38.6%)	88 (44.4%)	73 (40.6%)	32 (26.2%)	
Chronic LDL-C lowering therapy					
Prior statin	100 (20.0%)	30 (15.2%)	42 (23.3%)	28 (23.0%)	0.09
Prior ezetimibe	8 (1.6%)	2 (1.0%)	2 (1.1%)	4 (3.3%)	0.24
Baseline lipid profile					
Total cholesterol (mg/dL)	171 [147–199]	177 [154–201]	167 [141–194]	170 [143–203]	0.16
LDL-C (mg/dL)	109 [88–134]	107 [90–130]	106 [85–134]	114 [90–140]	0.17
HDL-C (mg/dL)	40 [34–48]	42 [36–49]	38 [32–45]	41 [34–49]	**0.002**
Triglycerides (mg/dL)	97 [68–135]	114 [84–145]	93 [63–128]	80 [58–116]	**<0.001**
Drugs at discharge					
ASA	500 (100.0%)	198 (100.0%)	180 (100.0%)	122 (100.0%)	N.A.
DAPT	498 (99.6%)	197 (99.5%)	180 (100.0%)	121 (99.2%)	0.52
ACE-inhibitors/Sartans	429 (85.8%)	173 (87.4%)	150 (83.3%)	106 (86.9%)	0.49
Beta-blockers	436 (89.3%)	169 (85.4%)	161 (89.4%)	106 (96.4%)	**0.011**

Period A: LDL-C goal <70 mg/dL; Period B: LDL-C goal <55 mg/dL and stepwise approach; and Period C: LDL-C goal <55 mg/dL and personalized, SES lipid-lowering approach. Values are expressed as *N* (%) or median [interquartile range]. ACE, angiotensin-converting enzyme; ASA, acetylsalicylic acid; BMI, body mass index; DAPT, dual antiplatelet therapy; HDL-C, high-density lipoprotein cholesterol; LDL-C, low-density lipoprotein cholesterol; LVEF, left ventricular ejection fraction; MI, myocardial infarction; NSTEMI, non-ST-segment elevation myocardial infarction; PCI, percutaneous coronary intervention; SES, strike early and strong; STEMI, ST-segment elevation myocardial infarction.

Significant *P* values are reported in bold.

**Figure 2:**
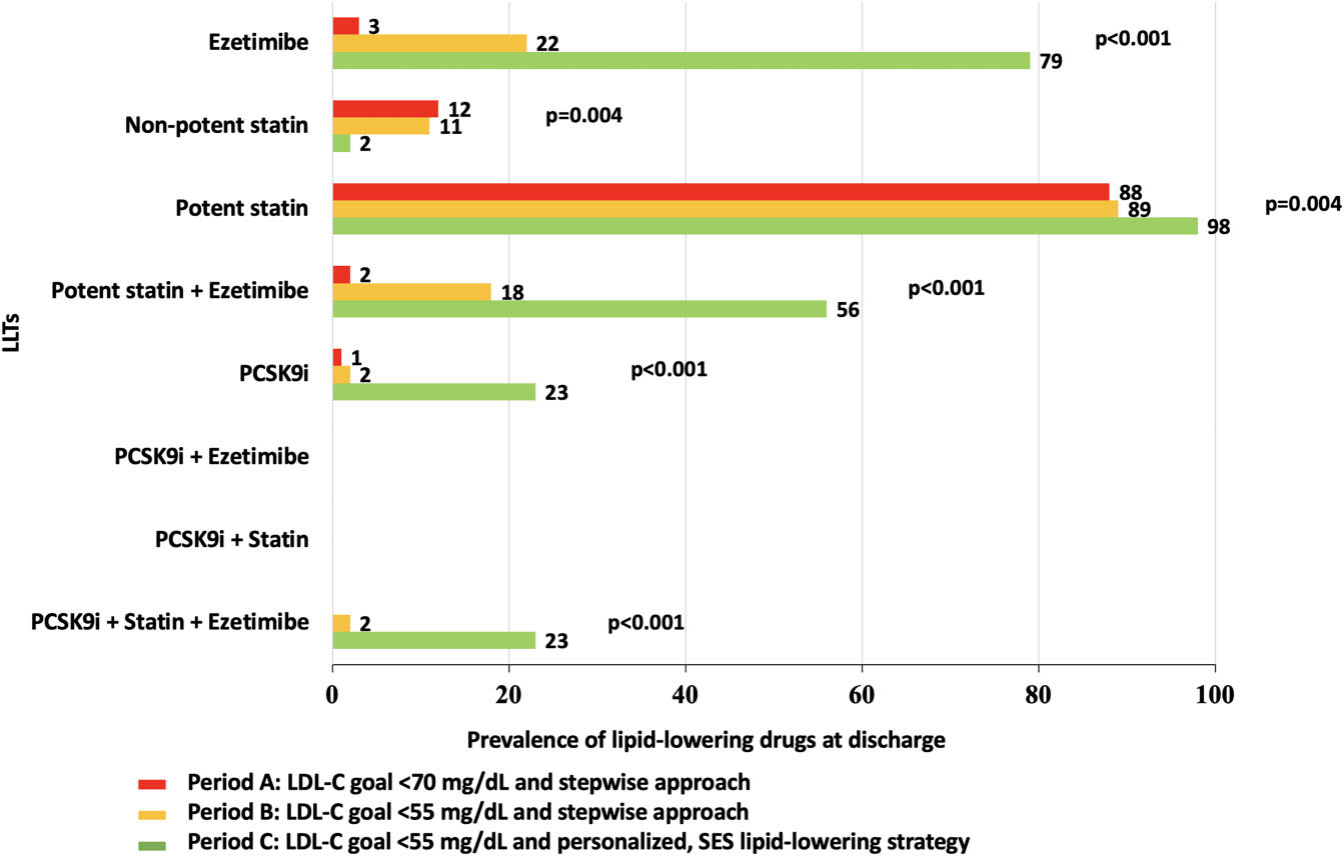
LLTs at discharge in the three periods. LDL-C, low-density lipoprotein cholesterol; LLTs, lipid-lowering therapies; PCSK9i, proprotein convertase subtilisin/kexin type 9 inhibitor; SES, strike early and strong.

LLTs at discharge are detailed in *Figure 2*. The prescription of a potent statin increased from 88% in Period A and 89% in Period B to 98% in Period C (*P* = 0.004); this trend was also present for the use of ezetimibe (from 3% and 22% to 79%, *P* < 0.001) and for the combination of potent statin plus ezetimibe (from 2% and 18% to 56%, *P* < 0.001). A PCSK9 inhibitor was given in 1% of patients in Period A vs. 2% in Period B and 23% in Period C (*P* < 0.001). Among patients receiving a PCSK9 inhibitor at discharge, 27 were given evolocumab and 1 alirocumab. Baseline LDL-C levels in patients receiving the combination statin plus ezetimibe were 110 [88–127] mg/dL, and in those treated with PCSK9 inhibitor were 152 [142–176] mg/dL.

### LDL-C changes

**Figure 3:**
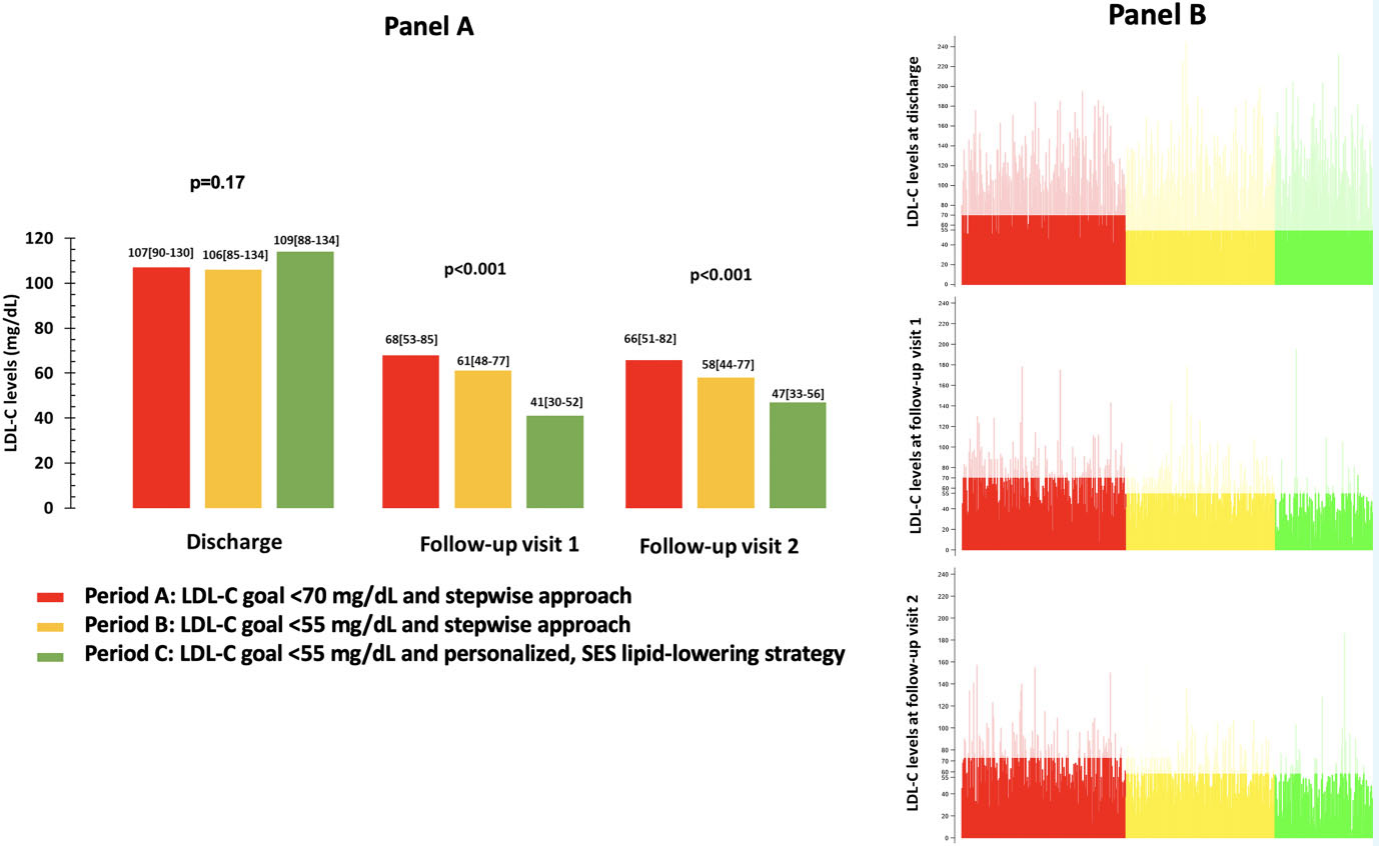
Panel A: Comparison of LDL-C levels between the three study periods at discharge, visit 1 and visit 2. Data are expressed as median [interquartile range]. Panel B: individual lowest LDL-C values at follow-up visits in the three periods. Levels of LDL-C at target are marked in evidence. LDL-C, low-density lipoprotein cholesterol; SES, strike early and strong.

Lipid values at follow-up visits are reported in Supplementary material online, *Table S1*. In Period A, LDL-C levels were reduced from discharge to visit 1 (−39%, *P* < 0.001) and did not change from visit 1 to visit 2. In Period B, the relative decrease of LDL-C concentrations from discharge to visit 1 was similar (−40%, *P* < 0.001), with values slightly reducing between the two follow-up visits. LDL-C levels in Period C had the greatest lowering from discharge to visit 1 (−60%, *P* < 0.001) and non-significantly increased from visit 1 to visit 2. For the comparisons between different periods, at both visits, LDL-C concentrations were lower in Period C vs. both Periods A and B (*Figure 3*, panel A). *Figure 3*, panel B visually represents the individual lowest LDL-C values at follow-up visits in the three periods.

**Figure 4:**
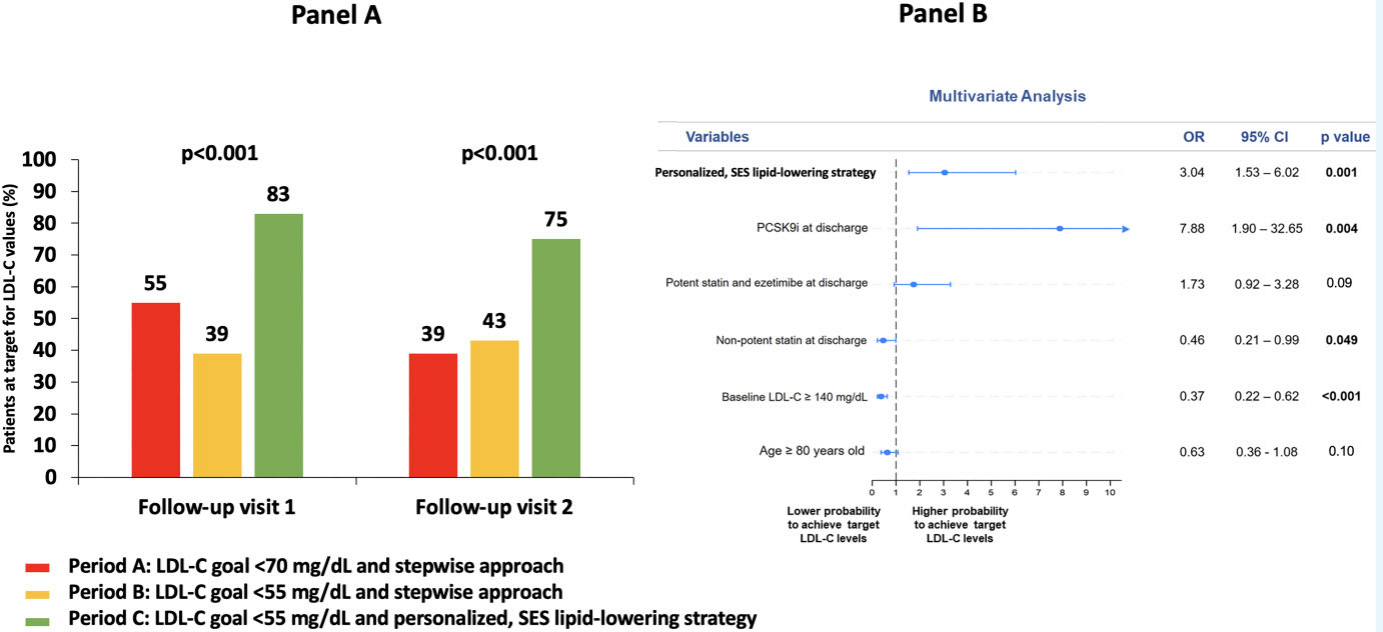
Panel A: Prevalence of patients achieving the LDL-C goal in at least one visit during follow-up. LDL-C goal was <70 mg/dL for Period A and <55 mg/dL for Period B and Period C. Panel B: Multivariate analysis for achieving the LDL-C goal in at least one visit during follow-up. LDL-C, low-density lipoprotein cholesterol; PCSK9i, proprotein convertase subtilisin/kexin type 9 inhibitor; SES, strike early and strong.

Consistently, regarding the laboratory primary endpoint, the proportion of patients achieving the recommended LDL-C goal at visit 1 was significantly higher in Period C (83%) vs. both Period A (55%) and Period B (39%), as depicted in *Figure 4*, panel A (*P* < 0.001), and this was maintained at visit 2: 75% vs. 39% and 43%, respectively (*P* < 0.001). Multivariate analysis (*Figure 4*, panel B) showed that the personalized, SES strategy for LLT was independently associated with a higher likelihood of achieving LDL-C goal in at least one visit during follow-up (adjusted OR: 3.04, *P* = 0.001). The use of PCSK9 inhibitors was associated with the greatest likelihood (aOR: 7.88, *P* = 0.004), whereas the use of non-potent statin at discharge and baseline LDL-C ≥140 mg/dL were predictors of a lower probability. Furthermore, the SES approach, the prescription of potent statin plus ezetimibe at discharge and the use of PCSK9 inhibitors were independently associated with a higher likelihood of achieving the LDL-C goal at both visits (Supplementary material online, *Figure S1*). Among patients enrolled in Period C, no difference in the achievement of the LDL-C goal was observed between those having different LDL-C concentrations at baseline (<115 mg/dL, essentially treated with statin monotherapy, vs. 115–149 mg/dL, mainly receiving statin plus ezetimibe, vs. ≥150 mg/dL, treated with triple LLT (Supplementary material online, *Table S2*).

As indicated before, LDL-C levels did not markedly change from visit 1 to visit 2. This occurred in all study periods and was particularly evident in Period C, when from visit 1 to visit 2, a total of 20% of patients received an escalation of LLT (increase of statin dose, change to potent statin, addition of ezetimibe, or a PCSK9 inhibitor), whereas 12% of patients had a de-escalation of LTT (decrease of statin dose, change to non-potent statin), mainly because of true or presumed statin-related side effects ( Supplementary material online, *Figure S2*). However, the prevalence of side effects attributable to LLTs between patients included in the three periods was similar (Supplementary material online, *Table S3*).

### Clinical outcomes

**Figure 5:**
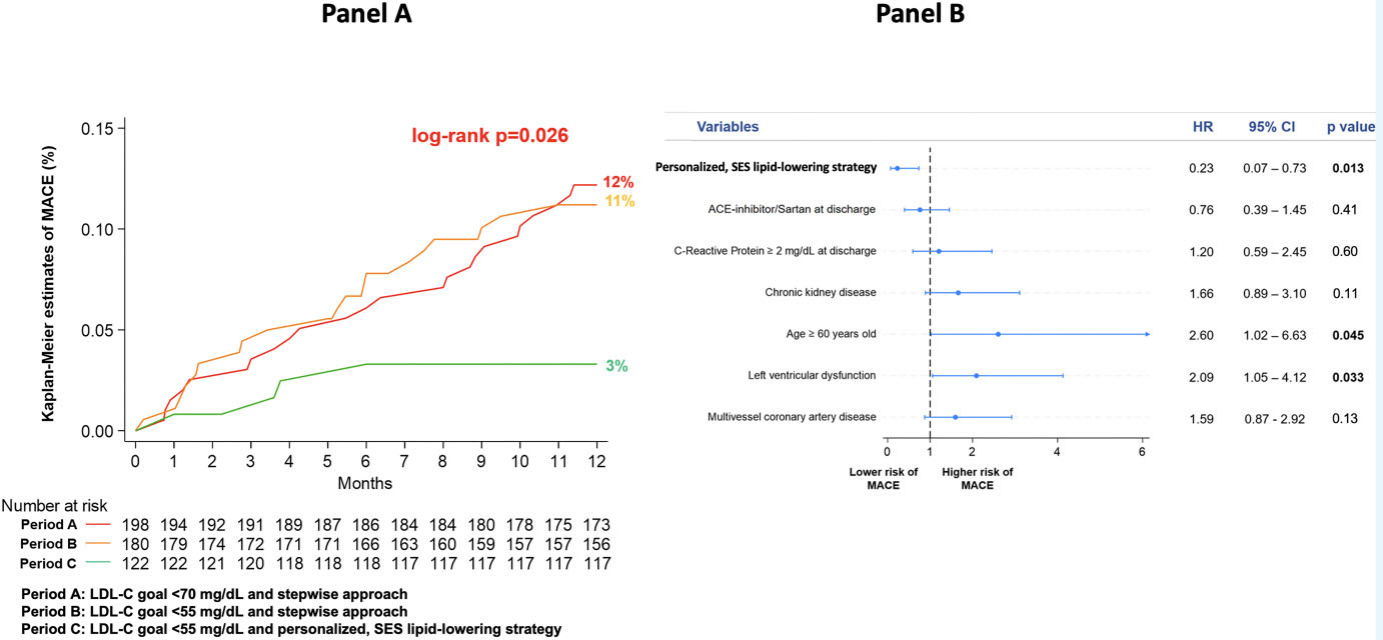
Panel A: Kaplan–Meier curves for the incidence of MACE at 1 year in the three periods. Panel B: Multivariate analysis for the risk of MACE at 1 year. This analysis highlights that the SES strategy was independently associated with significant reduction in the risk of MACE after adjusting for potential confounders and for variables with different prevalence among patients enrolled in different periods. Older age and left ventricular dysfunction were associated with increased risk. Left ventricular dysfunction was defined as left ventricular ejection fraction < 40%. Chronic kidney disease was defined as estimated glomerular filtration rate <60 mL/min/1.73 m^2^. ACE, angiotensin-converting enzyme; LDL-C, low-density lipoprotein cholesterol; MACE, major adverse cardiovascular events; SES, strike early and strong.

For the clinical primary endpoint, there was a similar 1-year incidence of MACE in Periods A and B (12% vs. 11%, *P* = 0.82), whereas a significantly lower MACE rate was observed in Period C (3%, global log-rank *P* = 0.026) (*Figure 5*, panel A). The occurrence of the individual components of the primary composite endpoint is reported in Supplementary material online, *Table S4*. Multivariate analysis showed that the SES strategy was independently associated with significant reduction in the risk of MACE after adjusting for potential confounders (*Figure 5*, panel B). The incidence of MACE in Period C was similar across different LLT strategies at discharge (Supplementary material online, *Table S5*).

**Figure 6:**
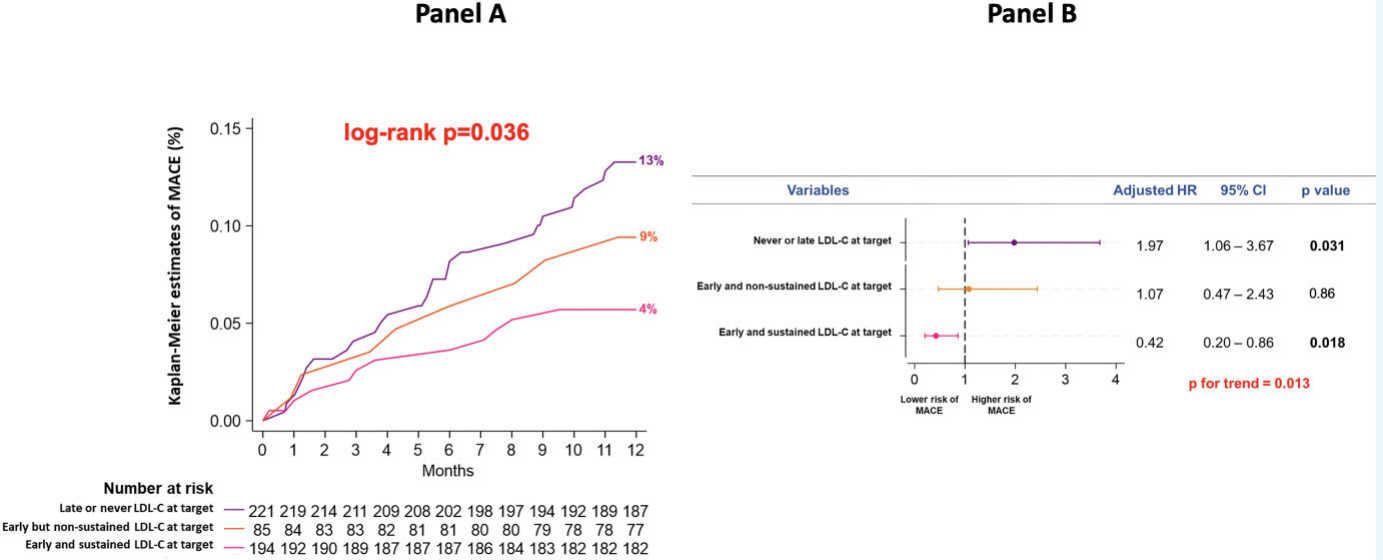
Panel A: Kaplan–Meier curves for the incidence of MACE at 1 year in patients achieving the LDL-C goal at both follow-up visits (early and sustained LDL-C at target), in those reaching the goal at visit 1, but not at visit 2 (early, but non-sustained LDL-C at target), and in those reaching the goal only at visit 2 or never reaching the goal (late or never LDL-C at target). Panel B: Multivariate analysis for the risk of MACE at 1 year in patients with early and sustained LDL-C at target, in those with early, but non-sustained LDL-C at target and in those with late or never LDL-C at target. LDL-C, low-density lipoprotein cholesterol; MACE, major adverse cardiovascular events.

In the overall study population, Kaplan–Meier analysis confirmed a better clinical outcome in patients reaching the goal of LDL-C <55 mg/dL in at least one follow-up visit (5% vs. 13% in those not achieving the goal, *P* = 0.006) (Supplementary material online, *Figure S3*). Of note, the incidence of MACE was the lowest in patients achieving the LDL-C goal at both follow-up visits (early and sustained LDL-C at target: 4%), intermediate in those with LDL-C at target at visit 1, but not at visit 2 (early, but non-sustained LDL-C at target: 9%), and highest in those reaching the LDL-C goal only at visit 2 or never achieving the goal (late or never LDL-C at target: 13%; log-rank *P* = 0.036) (*Figure 6*, panel A). At multivariate analysis, the risk of MACE was reduced in patients with early and sustained LDL-C at target (aHR 0.42, *P* = 0.018) and was increased in those with late or never LDL-C at target (aHR 1.97, *P* = 0.031); having early, but non-sustained LDL-C at target did not significantly impact on MACE occurrence (aHR 1.07, *P* = 0.86; *P* for trend = 0.013) (*Figure 6*, panel B).

Finally, the percentage of patients who during follow-up achieved both the goal of LDL-C <55 mg/dL and ≥ 50% LDL-C reduction from baseline was significantly higher in Period C [65% vs. 22% in Period A (*P* < 0.001) and 26% in Period B (*P* < 0.001)]. Importantly, in the subgroup reaching both the goal of LDL-C <55 mg/dL and ≥ 50% LDL-C reduction, the incidence of MACE was the lowest (Supplementary material online, *Figure S4*).

## Discussion

This study provides evidence that the systematic introduction in patients with acute MI of an SES strategy for LLT, aimed at completely optimizing the treatment during index hospitalization or at discharge, resulted in a substantial shift towards more effective and earlier LDL-C reduction. Importantly, this was associated with the lower risk of MACE at 1 year, due to higher rates of patients achieving the recommended <55 mg/dL LDL-C goal.

Our results on January–June 2019 and January–June 2021 periods, when the stepwise approach was recommended, indicate very low percentages of patients with acute MI receiving as LLT the combination of statin plus ezetimibe or the prescription of PCSK9 inhibitors. This feature carried a largely suboptimal achievement of the LDL-C goal, confirming data from various international registries,^9–17^ as well as a high incidence of MACE at 1 year. Such issue highlights a significant discrepancy between recommendations provided by the ESC/EAS guidelines on dyslipidemias and the results of their application in the real-world setting. Notably, even with the availability of more potent agents and combination treatments, the stepwise LLT approach in patients with acute MI appears ineffective. Indeed, the stepwise approach might work well, but in the real world, it has various limitations precluding its correct application: requires time; causes delay in LLT optimization and in clinical benefit; is often associated with physician's therapeutic inertia preventing its correct application, as well as with reduced patient's motivation to continue effective therapies for secondary cardiovascular prevention leading to poor adherence to treatment. Thus, simply lowering the LDL-C goals, without incorporating a structured protocol of SES for LLT, did not produce a significant clinical benefit. In our investigation, this is demonstrated by a similar occurrence of MACE in Periods A and B, when the LDL-C goals were different, but the stepwise approach was a common condition. Our results underscore the need for coupling the introduction of lower LDL-C goals with the implementation of comprehensive, individually tailored therapeutic strategies able to ensure these goals may be effectively reached. Furthermore, the stepwise approach may also present country-specific disadvantages, often preventing evidence-based and guideline-directed treatments, such as limited availability of repeated and timely follow-up visits, with consequent loss of those patients who are not seen on a regular basis; administrative barriers to drug prescription; delays in the incorporation of new drugs into regional formularies; and time-consuming processes of drug prescription.^18^

From January 2023, in patients with acute MI and LDL-C values above the recommended goal, we systematically adopted a predefined protocol of personalized SES for LLT, replacing the stepwise approach. This SES strategy was based on background chronic LLT, baseline LDL-C concentrations, % LDL-C reduction required to meet the goal, and expected LDL-C reduction with available LLTs. Cardiologists in our Institution have shared such therapeutic algorithm, where a complete LLT, able to potentially early achieve the LDL-C goal, was given already during in-hospital stay or at discharge. This led to potentiate the use of oral lipid-lowering strategies, e.g. potent statin at high dose in almost all patients and statin plus ezetimibe in > 50% of patients; moreover, a PCSK9 inhibitor was used in 23% of patients, e.g. only when the required LDL-C reduction was ≥ 45% in statin-treated patients and ≥ 65% in statin-naïve. Such approach was associated with a median LDL-C value of 41 mg/dL at the first follow-up visit, when 83% of patients gained the recommended <55 mg/dL LDL-C goal. Notably, with the personalized SES strategy, the attainment of the LDL-C goal was not significantly different among patients having baseline LDL-C <115 mg/dL (mainly receiving statin monotherapy), 115–149 mg/dL (essentially treated with statin plus ezetimibe) or ≥150 mg/dL (receiving triple LLT). Furthermore, the incidence of MACE at 1 year was not significantly different in patients discharged on statin monotherapy, statin plus ezetimibe, or triple LLT. Importantly, the occurrence of side effects attributable to the LLT was not increased in the SES period compared to the other periods. Of note, in our population multivariate analysis showed that SES strategy and use of PCSK9 inhibitors were associated with a three-fold and approximately an eight-fold increase, respectively, in the likelihood of achieving the LDL-C goal; use of non-potent statins at discharge and a baseline LDL-C ≥140 mg/dL carried a significantly lower likelihood, emphasizing the need for potent LLTs able to ensure a substantial reduction in LDL-C (‘strike strong’). Finally, we observed the lack of significant amelioration in the quality and intensity of LLT regimens across post-discharge follow-up visits. This underscores the clinical relevance of the ‘strike early’ approach, aimed at fully optimizing the LLT already during the hospitalization for the acute coronary event, and confirms that an earlier attainment of the LDL-C goal is a robust predictor of sustained lipid control.^11,20^

In our study the incidence of MACE at one year during the period of the personalized SES approach was significantly lower vs. the other periods; this occurred thanks to the achievement of the <55 mg/dL LDL-C goal in a great proportion of patients. The outcome improvement was greater when the achievement of the LDL-C goal was associated with ≥ 50% LDL-C reduction from baseline. Importantly, the decrease in the MACE risk was also more pronounced in patients with early and sustained LDL-C at target; of note, having an early, but non-sustained LDL-C at target (e.g. LDL-C goal achieved at follow-up visit 1, but not maintained at follow-up visit 2) did not reduce the risk and having late or never LDL-C at target increased the risk. Despite the abovementioned limitations, the 2023 ESC guidelines on acute coronary syndromes continued to recommend the stepwise approach for LLT (class IA).^21^ Such recommendation is based on a combination of long-term evidence and individual risk assessment, where starting with a statin and escalating subsequently to combination treatments may present a good cost-benefit ratio.^2^ In these guidelines, the use of LLTs in combination from the very beginning has a class IIb recommendation, as to date, the clinical evidence supporting the SES strategy is very limited.^21^ For the first time, our findings demonstrate that, as compared with the stepwise approach, the SES strategy is associated with better cardiovascular outcome, supporting that it can more effectively address the residual risk related to recurrent cardiovascular events. Recently, the multicenter, Italian, AT-TARGET-IT registry demonstrated the clinical benefit of a fast-track approach for LLT after an acute coronary syndrome, where a PCSK9 inhibitor was used in all patients.^22^ Moreover, data from the SWEDEHEART registry showed a lowest risk of cardiovascular events when the on-treatment non-HDL-C goal was early obtained after MI (within 2 months) and maintained thereafter.^23^ All these findings challenge the stepwise approach for LLT in patients with acute MI,^24^ which inevitably leads to delay in goal attainment or no goal achievement and possible harm.

Previous randomized data highlighted the clinical relevance of an immediate and intensive lipid-lowering approach in patients with acute coronary syndrome, regardless of the timing and type of treatment strategies.^25,26^ Recent studies using intracoronary imaging techniques provided the patho-physiological link supporting the benefit of intensive LLTs even over the short term. Here, a coronary plaque stabilization by optical coherence tomography analysis was demonstrated with rosuvastatin and with evolocumab on top of statin treatment after only 3 months in patients with stable coronary disease and acute coronary syndrome, respectively.^27,28^ Such beneficial effects on coronary plaques were confirmed over a longer term in two randomized trials, where a PCSK9 inhibitor (evolocumab in HUYGENS, alirocumab in PACMAN-AMI) was early initiated after an acute MI.^29,30^ An intensive LLT increases coronary plaque stability through various mechanisms^29–32^: decrease in the lipid content, leading to a reduction in plaque size and vulnerability; attenuation of macrophage infiltration within the plaque, which is linked to local inflammatory responses and may lower the risk of rupture and subsequent thrombus formation; improvement of endothelial function, favouring the repair of the endothelial layer and vascular health; and fibrous cap thickening, with reduced risk of future adverse events due to plaque rupture; mitigation of atherosclerosis progression, promoting long-term cardiovascular stability and improving patient's outcomes. Whether an upstream initiation of PCSK9 inhibitors in patients with acute MI improves cardiovascular outcomes will be clarified from the ongoing EVOLVE-MI (ClinicalTrials.gov: NCT05284747) and AMUNDSEN (ClinicalTrials.gov: NCT04951856) trials.

The present investigation has to be considered in light of its limitations inherent to the retrospective design. There were differences in patients’ characteristics between the study periods, reflecting the variability of the real-world clinical practice; however, the robustness of our findings was confirmed at multivariate analyses. An inclusion bias cannot be excluded, but it is unlike, as patients admitted for acute MI were consecutively enrolled. Moreover, the risk of residual confounding is present, but we found consistent results across different analyses. The investigation was conducted at a single center, which can affect the applicability of the findings to other institutions or healthcare systems. However, the longitudinal evaluation of outcomes according to over time changing practice patterns, performed within the same institution by the same cardiological staff, may represent a strength of the study. The difference in the number of patients enrolled across the three periods reflects the increased availability of 24-h cath-lab services from 2021 to 2023 in hospitals close to our center. The lack of a control group represents a limitation, as well as a certain degree of outcome improvement due to over time ameliorations of techniques and devices in interventional cardiology cannot be excluded. An ambulatory service focused on educational programs of secondary cardiovascular prevention was implemented at our center, but this occurred from 2019 and had no a differential impact across the study periods. Finally, the investigation was performed before the introduction in our institution of inclisiran and bempedoic acid.

In conclusion, the lack of structured LLT strategies is a recognized barrier preventing evidence-based treatments. We demonstrated that the systematic introduction of a personalized SES approach for LLT marked a relevant advancement in the management of patients with acute MI. The SES strategy was performed during hospitalization or at discharge and outperformed the traditional stepwise approach, by enabling a rapid and sustained achievement of LDL-C goals. This effect was mainly obtained through optimization of the use of potent statins at high dose and ezetimibe (e.g. generic drugs at low cost), while PCSK9 inhibitors were prescribed in less than one-fourth of patients; such issue indicates that our approach may be also sustainable in terms of health costs. Importantly, the application of the SES strategy translated into a significant reduction of recurrent cardiovascular events at 1 year; future researches are needed to specifically quantify its economic implications on healthcare systems and its cardiovascular benefit over the longer term.

## Supplementary Material

pvaf004_Supplemental_Files

## Notes

**Author Contributions:** G.P.: Concept and Design; L.G., L.C., M.B., A.M.: Acquisition of Data; All authors: Analysis and Interpretation of Data; G.P., L.G.: Drafting of the Manuscript; All authors: Critical Revision of the Manuscript for Important Intellectual Content; L.G., L.C.: Statistical Analysis; G.P. and L.G., Administrative, Technical, or Material Support; G.P.: Supervision*.* L.G., L.C., M.B., A.M. had full access to all of the data in the study and take responsibility for the integrity of the data and the accuracy of the data analysis.

## Data availability

No data available.
