# Supplementary material for: Impact of a personalized, strike early and strong lipid-lowering approach on low-density lipoprotein-cholesterol levels and cardiovascular outcome in patients with acute myocardial infarction
Source: Eur Heart J Cardiovasc Pharmacother. 2025 Jan 24;11(2):143–54. doi: 10.1093/ehjcvp/pvaf004 (PMC11905752; doi:10.1093/ehjcvp/pvaf004)
Supplement: pvaf004_Supplemental_Files [file pvaf004_supplemental_files.zip › Supplementary Table 1_OK.docx]

|  |  |  | Period |  |  |
| --- | --- | --- | --- | --- | --- |
|  |  | A | B | C | *p value* |
|  |  | N=198 | N=180 | N=122 |  |
|  |  |  |  |  |  |
| ***Lipid profile at visit 1*** |  |  |  |  |  |
| Total cholesterol (mg/dL) |  | 131 [114 - 148] | 119 [104.5 - 139] | 103 [87 - 116] | **<0.001** |
| LDL-C (mg/dL) |  | 68 [53 - 85] | 61 [48 - 77] | 41 [30.4 - 52] | **<0.001** |
| HDL-C (mg/dL) |  | 42 [34 - 48] | 39 [32 - 45] | 41 [33 - 49] | **0.016** |
| Triglycerides (mg/dL) |  | 98 [75 - 130] | 84 [64.5 - 124] | 87 [61 - 109] | **<0.001** |
|  |  |  |  |  |  |
| ***Lipid profile at visit 2*** |  |  |  |  |  |
| Total cholesterol (mg/dL) |  | 134 [114 - 151] | 120 [100 - 138] | 107 [90 - 122] | **<0.001** |
| LDL-C (mg/dL) |  | 66 [51 - 82] | 58 [45 - 77] | 47 [33 - 56] | **<0.001** |
| HDL-C (mg/dL) |  | 42 [35 - 52] | 41 [34 - 47] | 43 [35 - 52] | 0.07 |
| Triglycerides (mg/dL) |  | 101 [80 - 142] | 82 [64 - 112] | 86 [64 - 109] | **<0.001** |

**Supplementary Table 1.** Lipid values at follow-up visits in patients enrolled in the three periods.

Values are expressed as median [interquartile range]. HDL-C= High-Density Lipoprotein Cholesterol; LDL-C= Low-Density Lipoprotein Cholesterol. Significant p values are reported in bold.
