# Supplementary material for: Impact of a personalized, strike early and strong lipid-lowering approach on low-density lipoprotein-cholesterol levels and cardiovascular outcome in patients with acute myocardial infarction
Source: Eur Heart J Cardiovasc Pharmacother. 2025 Jan 24;11(2):143–54. doi: 10.1093/ehjcvp/pvaf004 (PMC11905752; doi:10.1093/ehjcvp/pvaf004)
Supplement: pvaf004_Supplemental_Files [file pvaf004_supplemental_files.zip › Supplementary Table 2.docx]

|  | Baseline LDL-C <115 mg/dL  N=62 | Baseline LDL-C 115–149 mg/dL  N=41 | Baseline LDL-C ≥150 mg/dL  N=19 | *p value* |
| --- | --- | --- | --- | --- |
| Patients with LDL-C at target during follow-up | 38 (61) | 32 (78) | 14 (74) | 0.18 |

**Supplementary Table 2.** Patients with LDL-C at target during follow-up according to different baseline LDL-C levels in the period C population (N=122). Values are expressed as number of patients (%). LDL-C= Low-Density Lipoprotein Cholesterol
