# Supplementary material for: Impact of a personalized, strike early and strong lipid-lowering approach on low-density lipoprotein-cholesterol levels and cardiovascular outcome in patients with acute myocardial infarction
Source: Eur Heart J Cardiovasc Pharmacother. 2025 Jan 24;11(2):143–54. doi: 10.1093/ehjcvp/pvaf004 (PMC11905752; doi:10.1093/ehjcvp/pvaf004)
Supplement: pvaf004_Supplemental_Files [file pvaf004_supplemental_files.zip › Supplementary Table 3.docx]

|  | Period | | | | |
| --- | --- | --- | --- | --- | --- |
|  | A | B | C |  | *p value* |
|  | N=198 | N=180 | N=122 |  |  |
|  |  |  |  |  |  |
| Overall side effects | 8 (4.0) | 11 (6.1) | 3 (2.5) |  | 0.30 |
| Muscular | 7 (3.5) | 1 (0.6) | 3 (2.5) |  |  |
| Gastrointestinal | 1 (0.5) | 9 (4.9) | 0 |  |  |
| Others | 0 | 1 (0.6) | 0 |  |  |
|  |  |  |  |  |  |

**Supplementary Table 3.** Side effects attributable to LLTs in patients enrolled in the three periods. Values are given as number of patients (%). LLT= Lipid-lowering therapy.
