# Supplementary material for: Impact of a personalized, strike early and strong lipid-lowering approach on low-density lipoprotein-cholesterol levels and cardiovascular outcome in patients with acute myocardial infarction
Source: Eur Heart J Cardiovasc Pharmacother. 2025 Jan 24;11(2):143–54. doi: 10.1093/ehjcvp/pvaf004 (PMC11905752; doi:10.1093/ehjcvp/pvaf004)
Supplement: pvaf004_Supplemental_Files [file pvaf004_supplemental_files.zip › Supplementary Table 4.docx]

|  |  |  | Period |  |  |
| --- | --- | --- | --- | --- | --- |
|  | Overall population | A | B | C | *p value* |
|  |  |  |  |  |  |
|  | N=500 | N=198 | N=180 | N=122 |  |
|  |  |  |  |  |  |
| MACE | 48 (9.6) | 24 (12.1) | 20 (11.1) | 4 (3.3) | **0.023** |
| Non-fatal MI | 35 (7.0) | 18 (9.1) | 13 (7.2) | 4 (3.3) |  |
| Non-fatal stroke | 2 (0.4) | 1 (0.5) | 1 (0.6) | 0 |  |
| Unplanned coronary revascularization | 36 (7.2) | 19 (9.6) | 13 (7.2) | 4 (3.3) |  |
| Cardiovascular death | 4 (0.8) | 0 | 4 (2.2) | 0 |  |
|  |  |  |  |  |  |

**Supplementary Table 4.** MACE at one-year follow-up in the overall population and in patients enrolled in the three periods. Values are expressed as number of patients (%). MACE= Major adverse cardiovascular events; MI= Myocardial infarction. Significant p values are reported in bold.
