# Supplementary material for: Impact of a personalized, strike early and strong lipid-lowering approach on low-density lipoprotein-cholesterol levels and cardiovascular outcome in patients with acute myocardial infarction
Source: Eur Heart J Cardiovasc Pharmacother. 2025 Jan 24;11(2):143–54. doi: 10.1093/ehjcvp/pvaf004 (PMC11905752; doi:10.1093/ehjcvp/pvaf004)
Supplement: pvaf004_Supplemental_Files [file pvaf004_supplemental_files.zip › Supplementary Table 5.docx]

|  | LLT at discharge | | | | |
| --- | --- | --- | --- | --- | --- |
|  | Statin monotherapy  N=25 | Statin + ezetimibe  N=69 | Triple LLT  N=28 |  | *p value* |
|  |  |  |  |  |  |
| LDL-C at target during follow-up | 13 (52.0) | 49 (71.0) | 22 (78.6) |  | 0.10 |
|  |  |  |  |  |  |
| MACE incidence at one-year | 1 (4.0) | 2 (2.9) | 1 (3.6) |  | 0.96 |
|  |  |  |  |  |  |
|  |  |  |  |  |  |

**Supplementary Table 5.** Achievement of the LDL-C goal and MACE incidence according to different LLTs at discharge in patients enrolled in Period C. Values are expressed as number of patients (%). LDL-C= Low-Density Lipoprotein Cholesterol; LLT= Lipid-lowering therapy; MACE= Major adverse cardiovascular event.
