# Supplementary figures and images for: Impact of a personalized, strike early and strong lipid-lowering approach on low-density lipoprotein-cholesterol levels and cardiovascular outcome in patients with acute myocardial infarction
Source: Eur Heart J Cardiovasc Pharmacother. 2025 Jan 24;11(2):143–54. doi: 10.1093/ehjcvp/pvaf004 (PMC11905752; doi:10.1093/ehjcvp/pvaf004)

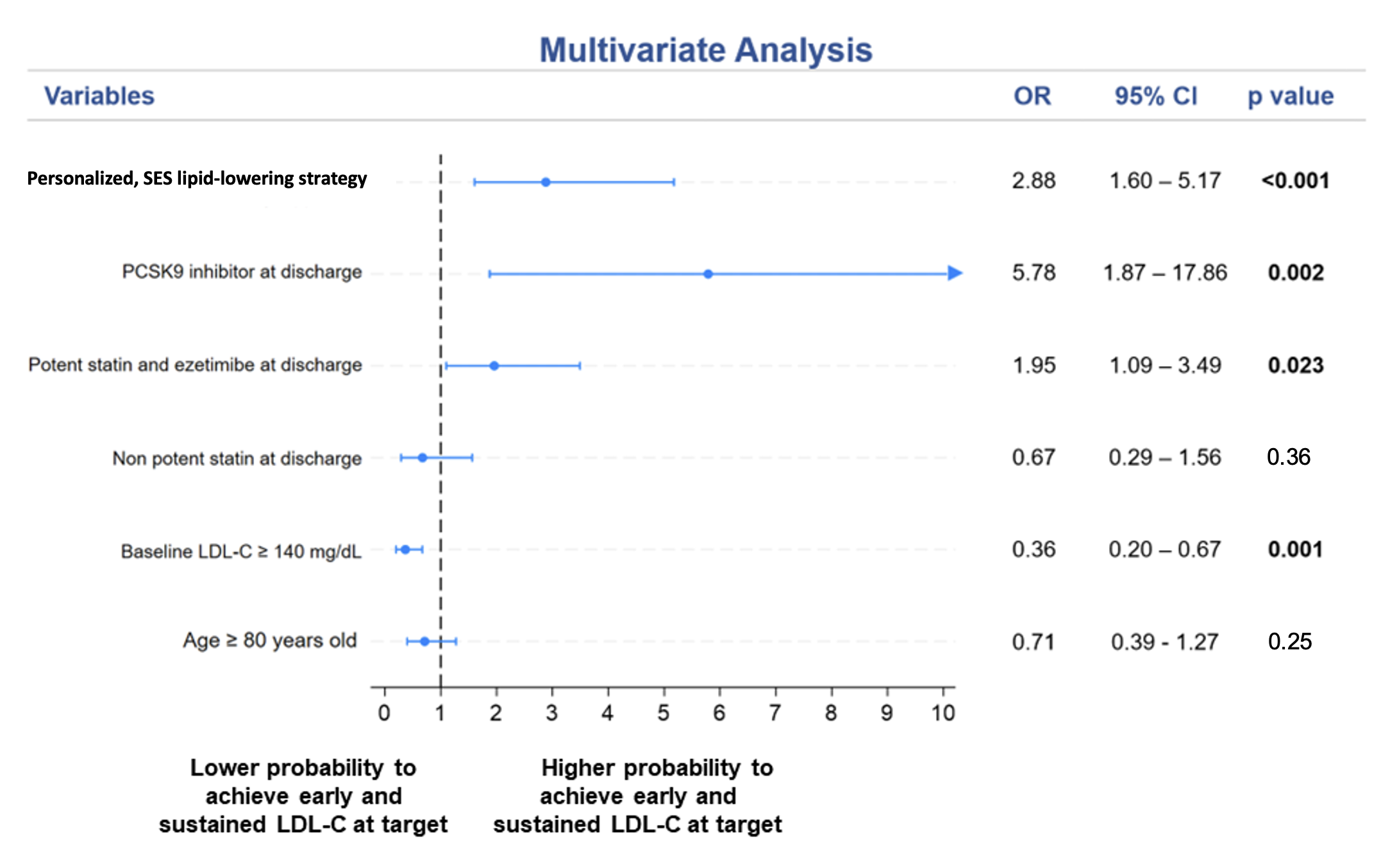

Supplement: pvaf004_Supplemental_Files [file pvaf004_supplemental_files.zip › Supplementary Figure 1.tiff]

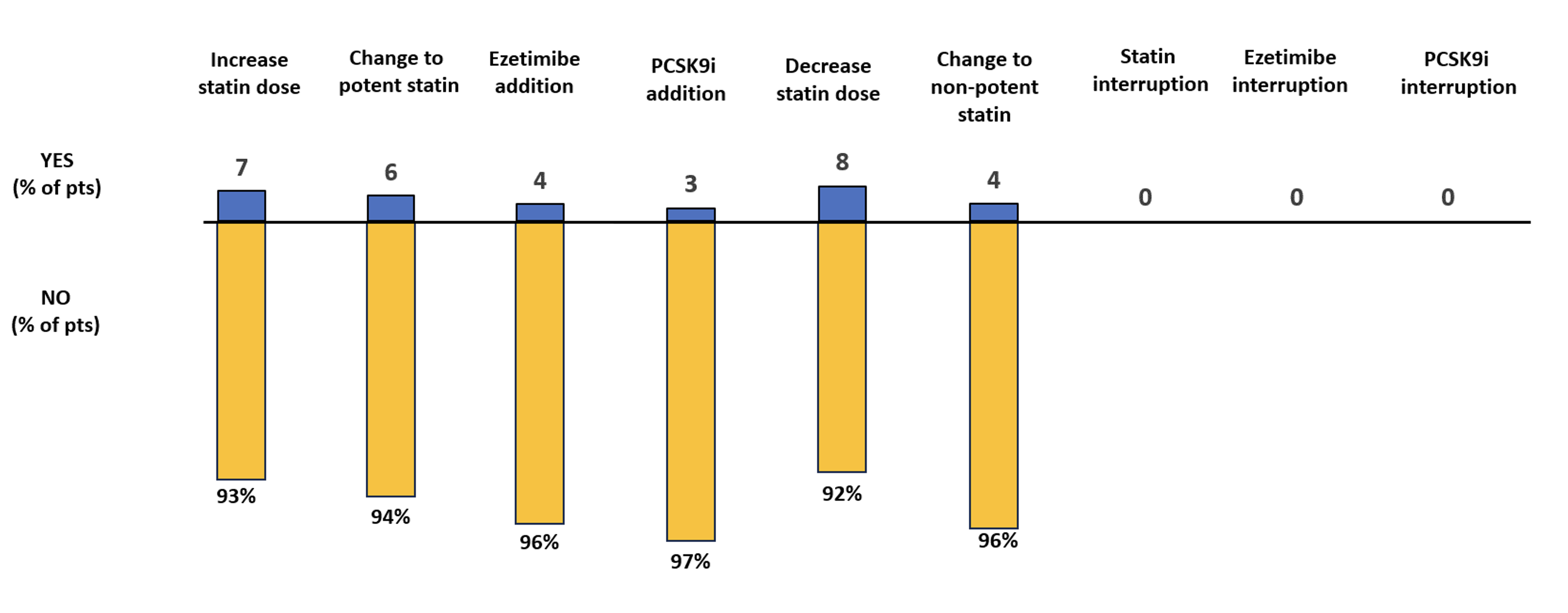

Supplement: pvaf004_Supplemental_Files [file pvaf004_supplemental_files.zip › Supplementary Figure 2.tiff]

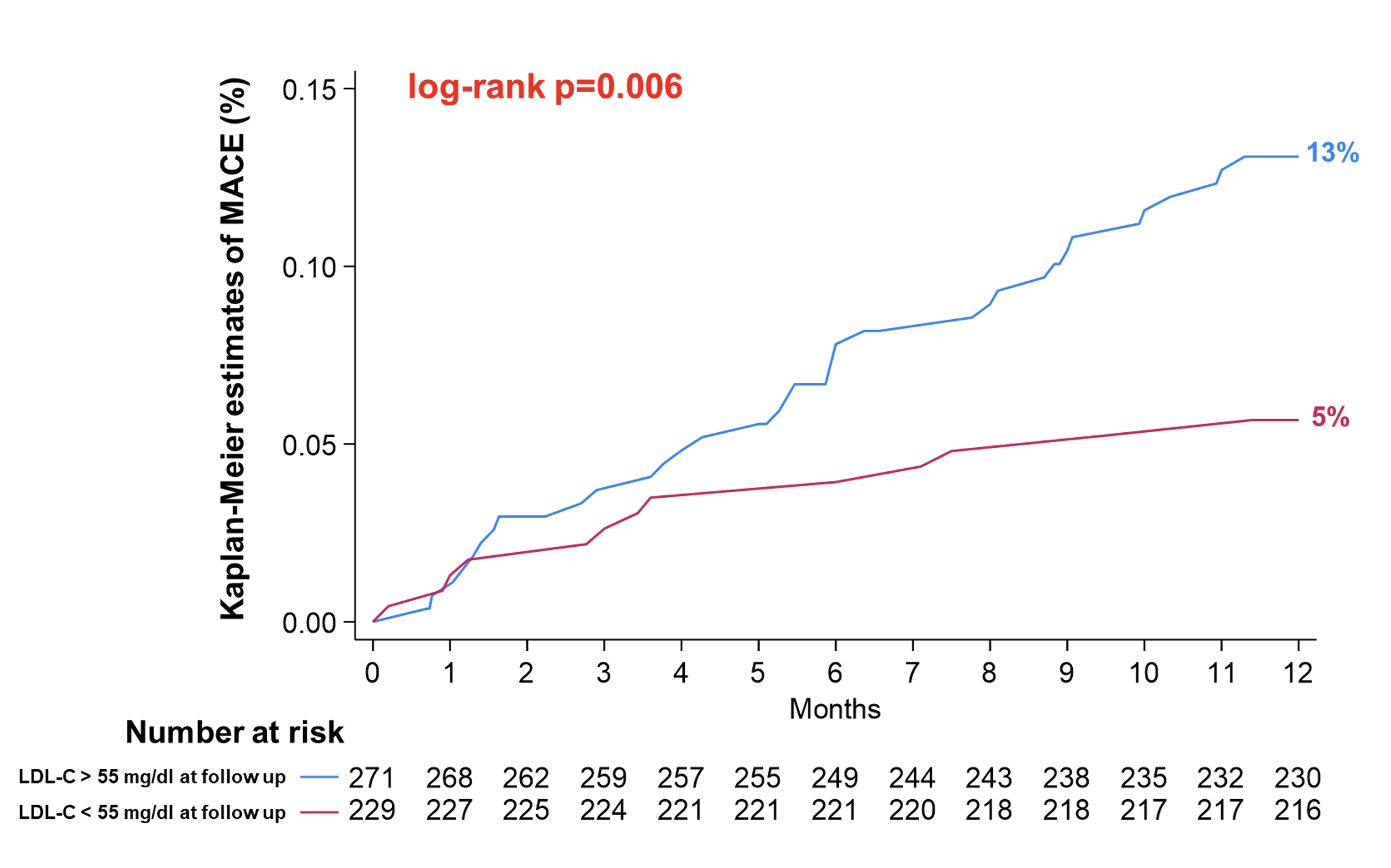

Supplement: pvaf004_Supplemental_Files [file pvaf004_supplemental_files.zip › Supplementary Figure 3.tiff]

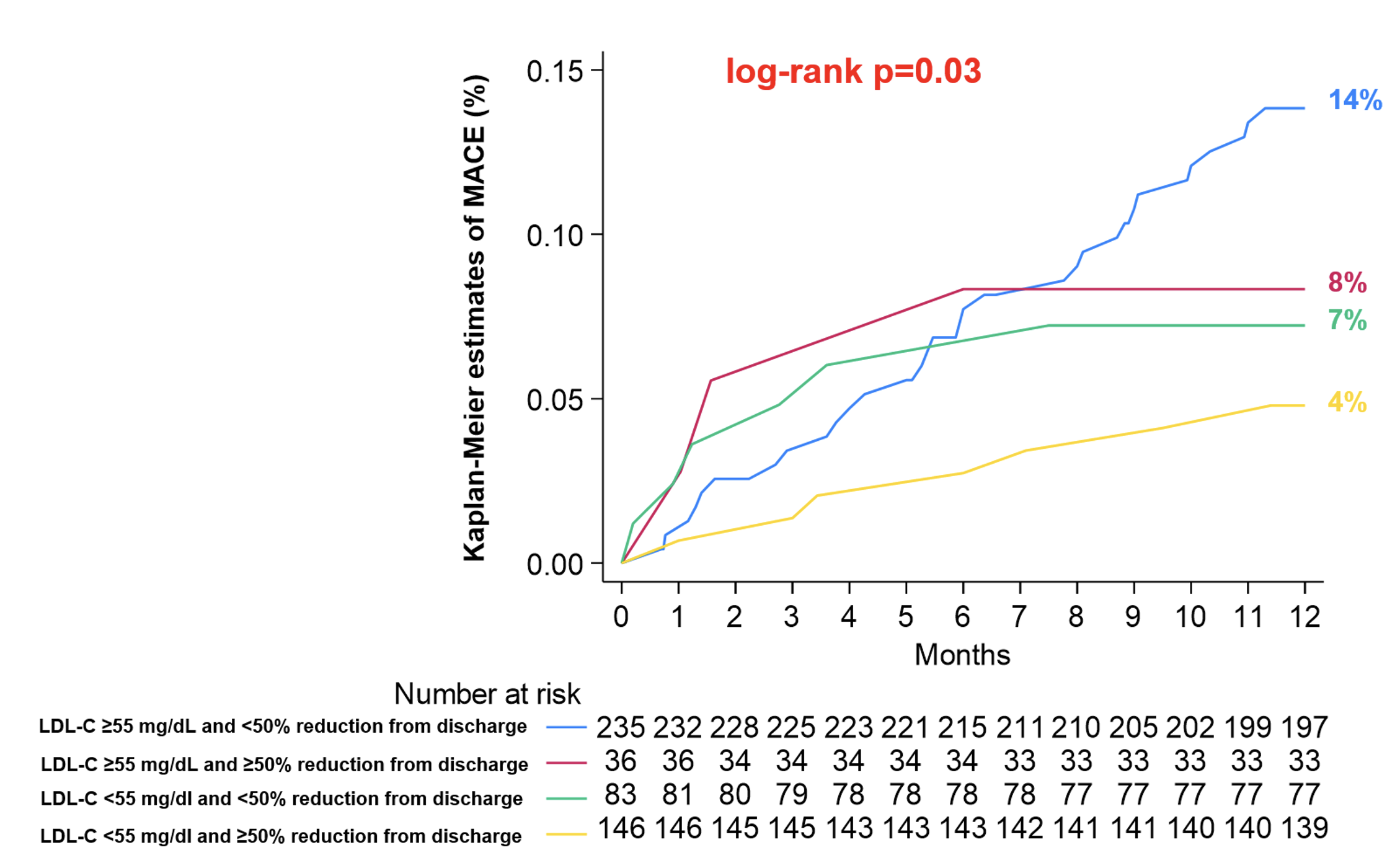

Supplement: pvaf004_Supplemental_Files [file pvaf004_supplemental_files.zip › Supplementary Figure 4.tiff]
